# Laboratory evaluation of higher-order aberrations and light scattering in explanted opacified intraocular lenses

**DOI:** 10.1186/s40662-021-00235-5

**Published:** 2021-04-22

**Authors:** Grzegorz Łabuz, Timur M. Yildirim, Gerd U. Auffarth, Hyeck-Soo Son, Ramin Khoramnia

**Affiliations:** David J Apple Center for Vision Research, Department of Ophthalmology, University of Heidelberg, Im Neuenheimer Feld 400, 69120 Heidelberg, Germany

**Keywords:** Higher-order aberrations, Intraocular lens, Straylight, IOL opacification

## Abstract

**Background:**

Intraocular lens (IOL) calcification is a serious condition that can only be treated by removing the clouded lens. Since explantation bears the risk of complications, it is often deferred until the patient finds the symptoms intolerable. Usually, as the IOL opacifies, visual acuity is minimally affected early on. In this study, we assessed the impact of IOL opacification on optical quality.

**Methods:**

We analyzed ten opacified explanted IOLs (Oculentis GmbH). Wavefront aberrations were obtained with a SHSOphthalmic device (Optocraft GmbH), which features a Hartmann-Shack sensor. The root mean square (RMS) of higher-order aberrations (HOAs) was compared. The effect of calcification on image quality was assessed through the Strehl ratio (SR). We detected light scattering with a C-Quant (Oculus GmbH) and expressed it as a straylight parameter.

**Results:**

At 2 mm, 3 mm and 4 mm, the mean RMS (±standard deviation) was 0.033 μm (±0.026 μm), 0.044 μm (±0.027), and 0.087 μm (±0.049), respectively. The mean SR value was 0.81 ± 0.15 at 3 mm, with four IOLs showing a nearly diffraction-limited performance, but in two explants, opacification precluded reliable measurements. Increased straylight was found in all opacified IOLs with a mean value of 150.2 ± 56.3 deg^2^/sr at 3 mm.

**Conclusions:**

We demonstrated that IOL opacification induces HOAs. However, the RMS remained low, which resulted only in a slight reduction of the SR-derived optical quality. On the other hand, we found a severe straylight elevation in the opacified lenses, which may result in dysphotopsia, such as glare, and subjective complaints, despite good visual acuity.

## Background

Materials used for intraocular lens (IOL) manufacturing are continually being evolved to improve their optical and biocompatibility properties while providing long-term clarity. Complications that develop after IOL implantation can depend directly on the constituent polymer material of the IOL. A condition such as late-postoperative precipitation of calcium phosphate crystals within the IOL material or on its surface is chiefly associated with IOLs made from hydrophilic acrylic copolymer [1–7]. When this relatively rare complication occurs after uneventful cataract surgery, and without subsequent surgery or other external factors, it is termed primary IOL calcification [8], and it is presumed to originate from the production or packaging of the lens, and thus it is episodic, limited to IOLs from a specific time of production and from one manufacturing site. Accordingly, in the past 25 years, the reports of serial explantations of calcified IOLs come from a succession of manufacturers including the Hydroview (Bausch & Lomb, USA), SC60B-OUV (Medical Developmental Research, USA) and Aqua-Sense (Ophthalmic Innovation International, Inc., USA) IOL models [1, 3]. The most recent series was reported in Lentis IOLs (Oculentis GmbH, Germany) [5, 6].

The only currently available treatment option in the case of IOL calcification is the surgical removal of the opaque IOL, and in most cases, with subsequent implantation of a new lens. The explantation of a calcified IOL, often many years after the initial cataract surgery, can lead to subsequent complications [9]. It is, therefore, essential to judge the patients’ visual impairment to advise them on the benefits and limitations of such a procedure.

Patients with IOL calcification typically report disturbing dysphotopsia, which are difficult to quantify [7, 10]. There is no single metric that can measure a patient’s impairment objectively. The way a calcified IOL induces visual disturbances is not well defined. Standardized functional metrics used in the clinic to assess a patient’s visual quality are visual acuity (VA), contrast sensitivity (CS), and less commonly, the quantification of intraocular straylight. While VA and CS do not seem to adequately describe the amount of impairment in patients with IOL calcification, a straylight meter can be used to quantify intraocular light scattering induced by this pathology [7].

Higher-order aberrations (HOAs) describe the distortion acquired by a wavefront of light when it passes through an imperfect optical system. Several commercially available devices offer the possibility to measure HOAs [11–20]. A high correlation was found between metrics derived from wave aberrations (e.g., visual Strehl ratio (SR)) and VA and subjective refraction [11, 13]. HOAs have also been used to describe symptoms like glare, halos, starburst, or double vision in different ocular conditions, such as dry eye [20] or post-laser in situ keratomileusis (LASIK) [17, 19]. The assessment of HOAs in patients suffering from dysphotopsia demonstrated a clear association of wave aberrations with reported symptoms [17, 19]. In one study, a two-fold increase in HOAs from the level found in asymptomatic patients resulted in an increased risk of visual disturbance [17]. Although the expectation is that media opacities may cause an increased level of (micro) aberrations [21], studies on HOAs in cases with IOL pathologies have not been reported so far.

This study aimed to compare different optical parameters, including HOAs, to find a suitable measure that quantifies the impact of IOL opacification on the patient’s visual impairment.

## Methods

### IOLs

We selected ten explanted IOLs that had been sent in 2018 and 2019 to the David J. Apple International Laboratory for Ocular Pathology (Heidelberg, Germany). In all cases, primary opacification was reported as the only reason for explantation. Table 1 presents the characteristics of affected patients provided by explanting surgeons from various centers. Photographs of IOL explants were obtained using a BX5 (Olympus Optical Co. Ltd., Japan) microscope. Although all the study lenses were from one manufacturer (Oculentis GmbH, Germany), four different models can be identified, which differ in their haptic configuration and the management of spherical aberration (Table 2). A clear + 20 D L-313 lens from the same manufacturer was used as a control.

**Table 1:** Demographic data of the study cases

Case no.	IOL model	Labeled power (D)	Date of implantation	Date of explantation	Age^**b**^ (years)	VA (preop)	VA (postop)	Microscopic images
**1**	LS-312-1 Y asph	21.5	12.01.2011	18.07.2018	81	0.5	0.5	
**2**	LS-313	23.2^a^	2012	28.06.2018	81	0.1	0.4	
**3**	LS-502-1	25.5	09.01.2012	26.06.2018	78	NA	NA	
**4**	L-313	19.5	17.11.2014	18.01.2019	67	0.16	0.1	
**5**	LS-502-1	19.5	21.02.2011	03.01.2019	80	0.9	NA	
**6**	L402	24.4^a^	NA	08.05.2019	49	1.25	0.9^d^	
**7**	L-312	21.9^a^	07.03.2017	02.05.2019	83	0.63^c^	0.3^c^	
**8**	L-402	18.0	12.10.2010	20.02.2019	68	1.0	NA	
**9**	LS-502-1	19.5	23.01.2012	07.05.2019	76	0.6	NA	
**10**	LS-402	23.0	12.12.2011	12.02.2019	86	NA	NA	
**Control**	L-313	20.0						

*IOL=* intraocular lens, *VA=* visual acuity, *NA=* not available

^a^The power measured by a wavefront device as the labeled power was not available;

^b^Patient’s age at explantation;

^c^Uncorrected visual acuity;

^d^Visual acuity obtained 1 day after surgery

**Table 2:** The comparison of studied intraocular lens models

**Model**	**LS-312-1 Y**	**L-313**	**LS-502-1**	**L402**
**Material**	Hydrophilic with a hydrophobic surface			
**Refractive index**	1.46			
**Water content**	25%			
**SA design**	Aberration Neutral		Spherical	
**Optic type**	Biconvex – aspheric posterior		Biconvex	
**Optic size (mm)**	6			
**Total size (mm)**	12	11	12	13
**Haptic design**	C-loop (1-piece)	Plate	C-loop (1-piece)	C-loop (3-piece)

*SA=* spherical aberration

### Wavefront assessment

Optical aberrations were obtained with a SHSOphthalmic device (Optocraft GmbH, Germany) for single-pass wavefront measurements and two repetitions. A light-emitting diode source with a center wavelength of 540 ± 10 nm was used. The light was projected through a collimator onto the test lens, located in a glass cuvette. Throughout measurements, the IOL was submerged in a balanced salt solution with a refractive index of 1.336. A visual inspection of lens alignment in the optical setup was made using a control camera, providing a continuous preview of the cuvette and the IOL. The 540-nm test beam passing through the IOL was imaged by a telescope onto a Hartmann-Shack device (SHSCam HR-110-GigE, Optocraft GmbH, Germany) with a size of 8.9 mm × 6.6 mm. The sensor features a 60 × 60 microlens system with an array pitch of 110 μm corresponding to a quadratic region of interest on a circular pupil.

Wavefront analysis was performed using the integrated software (SHSWorks, Optocraft GmbH, Germany). Zernike coefficients were computed up to the 6th order with a 2-, 3- and 4-mm mask and exported using Noll’s notation [22]. Although, in principle, the wavefront device can assess the full diameter of a clear IOL optic, in practice, the dense opacification located at the optic periphery in most of the study lenses precluded reliable measurements at higher apertures. The root mean square (RMS) of HOAs (including primary astigmatism but without defocus) was compared as well as the magnitude of astigmatism, coma, trefoil and spherical aberration up to the 6th order. The refractive power of the IOLs was obtained from wavefront measurements. In addition, the point spread function (PSF) of the IOL was derived from optical-aberration measurements and normalized by the diffraction-limited PSF. The modulation transfer function (MTF) was calculated to assess the impact of HOAs on image quality graphically. Besides, the SR was obtained in the frequency domain, which was estimated up to 100 lp/mm for sagittal (s) and tangential (t) MTFs and the normalization factor (theoretical diffraction-limited MTFs) according to the following formula [13].

$$ \mathrm{Strehl}\ \mathrm{ratio}=\frac{\iint_{-\infty}^{\infty}\mathrm{MTF}\left({\mathrm{f}}_{\mathrm{s}},{\mathrm{f}}_{\mathrm{t}}\right)\mathrm{d}{\mathrm{f}}_{\mathrm{s}}\mathrm{d}{\mathrm{f}}_{\mathrm{t}}}{\iint_{-\infty}^{\infty }{\mathrm{MTF}}_{\mathrm{t}\mathrm{heoretical}}\left({\mathrm{f}}_{\mathrm{s}},{\mathrm{f}}_{\mathrm{t}}\right)\mathrm{d}{\mathrm{f}}_{\mathrm{s}}\mathrm{d}{\mathrm{f}}_{\mathrm{t}}} $$

MATLAB (MathWorks Inc., Natick, MA, USA) was used for data analysis and visualization of amplitude of the HOAs.

### Straylight assessment

Light scattering in the explanted lenses was assessed with the C-Quant (Oculus GmbH, Germany), and a custom-designed adaptation for in vitro evaluation of IOLs [23–26]. In short, the straylight meter, which is a clinical device, consists of a test field subtending 3.3° of the visual angle in diameter, which is surrounded by a flickering annulus with inner and outer radius of 5° and 10°, respectively [23–26]. During clinical examination, both halves of the test field appear to flicker because the light originating from the annulus is scattered by the eye media toward the fovea. However, one half features compensation light to counter the flickering caused by scatter, as seen in the other half. A patient compares both halves and indicates which part of the test field flickers stronger using a two-alternative (left/right button) forced-choice paradigm. Based on the known level of the compensation light, patient’s responses are fitted using a psychometric function from which ocular straylight is determined according to this formula [27]:

$$ \mathrm{s}={\uptheta}^2\times \mathrm{PSF}\left(\uptheta \right)\left[{\deg}^2/\mathrm{sr}\right] $$

where θ is the effective visual angle of the flickering annulus (approx. 7.0°), and the PSF is a directly measured point spread function.

Although the C-Quant’s primary application is to measure ocular straylight in patients, the implementation of additional optical components allowed for an in vitro straylight evaluation of IOLs [23, 24]. In this adaptation, a test IOL was placed in a holder having a 3 mm diameter opening. Then, the lens and holder were inserted into a quartz cuvette filled with a balanced salt solution and positioned behind a converging lens. The positive element and its distance to the test IOL mimics an incidence angle established from in vivo assessment [23]. To the rear of the IOL, an adjustable diaphragm was placed and a magnifying lens that allows the test field to become visible to an observer irrespective of the IOL’s high refractive power. After loading and carefully aligning the test IOL into the apparatus, one manually adjusts the diaphragm size, and this effectively blocks the light originating from the flickering annulus, preventing it from reaching the observer’s eye. Therefore, we only measured scattering from the IOL following a procedure similar to the clinical evaluation’s psychophysical method. Two measurements per lens were taken. Only those with good reliability were used, which was judged based on the C-Quant’s quality parameter, i.e., estimated standard deviation (esd).

Measured straylight values were compared with normative data from the medical literature. The straylight parameter of an isolated crystalline lens was calculated for 20- and 80-year-old healthy eyes as well as that for a cataractous lens [28, 29], which was the average of the straylight parameter measured in three types of cataract (i.e., cortical, nuclear and posterior subcapsular) [29].

## Results

**Fig. 1:**
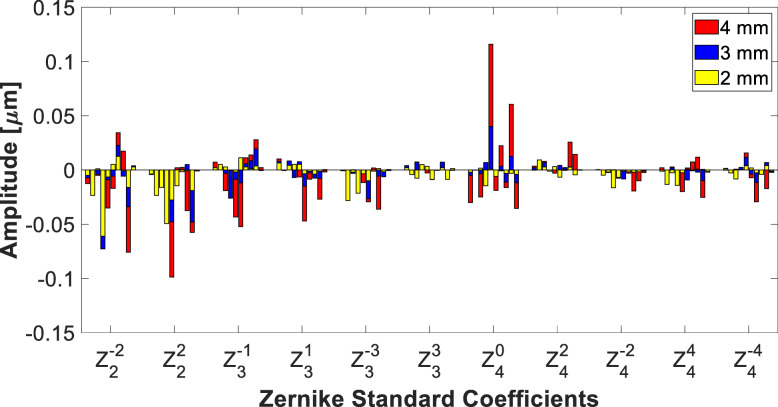
Zernike coefficients measured at 2-, 3- and 4-mm zone up to 4th order. The height of each color bar represents the measured amplitude. For each coefficient, one bar corresponds to one sample IOL with the order from IOL#2 to IOL#10 (from left to right). For each Zernike coefficient, the last bar corresponds to the control lens. At 2 mm (yellow), nine IOLs could be measured reliability (from IOL#2 to IOL#10). At 3 mm (blue), it was eight (without IOL#3), and seven (without IOL#5) at 4 mm (red) due to severe peripheral opacification

Figure 1 compares the amplitude of Zernike coefficients of the test IOLs at 2-, 3-, and 4-mm apertures. Because of dense opacification affecting the paracentral areas of the explanted IOLs, not all of them could be measured entirely at every aperture. One lens, IOL#1, was excluded as its light transmission was reduced by the opacity to such an extent that the device was unable to function at any aperture size. Thus, at 2 mm, the wavefront was measured in nine IOLs. At 3- and 4-mm aperture, aberrations were measured only in eight and seven IOLs, further eliminating IOLs #3 and #5, respectively.

HOAs of the control lens were close to zero besides spherical aberration, which was (virtually) the only measurable aberration at 3 mm and 4 mm. The calcified IOLs demonstrated varying levels of wavefront errors, including at the 2-mm mask. Astigmatism appears to be the dominant aberration showing the highest RMS irrespective of the aperture used, which was followed by coma and trefoil. Table 3 presents the RMS of all as well as the four types of HOAs.

**Table 3:** Wavefront aberrations up to the 6th order measured in the explanted and control IOLs at three pupil sizes

	2 mm				3 mm				4 mm			
	Opacified		Control		Opacified		Control		Opacified		Control	
	Mean	SD	Mean	SD	Mean	SD	Mean	SD	Mean	SD	Mean	SD
**Ast (μm)**	0.025	0.023	0.003	<1e-4	0.032	0.026	0.004	< 0.001	0.048	0.039	0.004	<1e-4
**Coma (μm)**	0.009	0.006	0.002	<1e-4	0.015	0.008	0.001	< 0.001	0.035	0.020	0.003	<1e-4
**Trefoil (μm)**	0.012	0.011	0.002	<1e-4	0.011	0.009	0.002	< 0.001	0.015	0.015	0.001	<1e-4
**SA (μm)**	0.005	0.005	0.004	<1e-4	0.012	0.012	0.012	< 0.001	0.045	0.034	0.033	0.002
**Total HOAs (μm)**	0.033	0.026	0.007	<1e-4	0.044	0.027	0.013	< 0.001	0.087	0.049	0.034	0.002

*SD=* standard deviation, *Ast=* astigmatism, *SA=* spherical aberration, *HOAs=* higher order aberrations (including ${Z}_2^{-2}$ and ${Z}_2^2$)

**Fig. 2:**
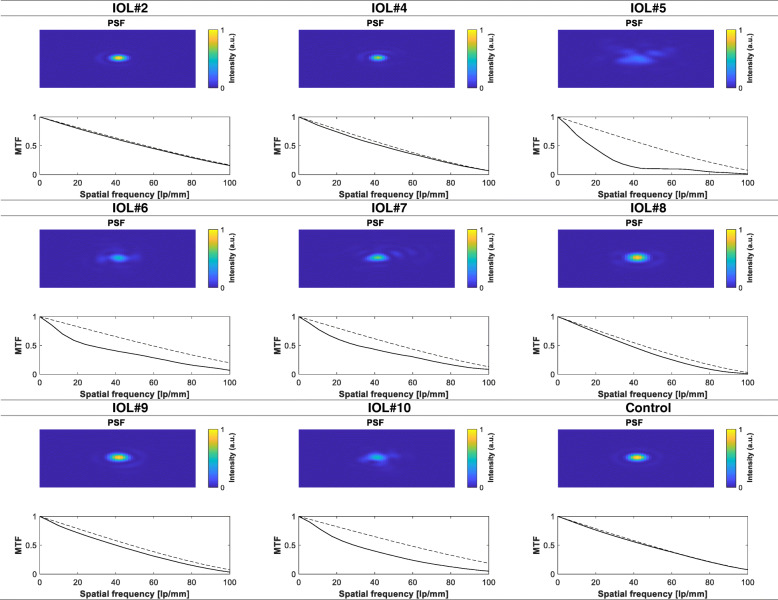
The modulation transfer function (MTF) and the point spread function (PSF) of the studied IOLs derived from wavefront aberrations at 3 mm. The dashed line is a diffraction-limited curve. Of the ten opacified lenses, measurements were possible in eight cases

The impact of wavefront aberration produced by IOL opacification on the PSF and the MTF at 3 mm are presented in Fig. 2. The optical quality was nearly diffraction-limited in the control IOL (SR = 0.98) and four explanted cases (IOLs #2, #4, #8 and #9) despite the presence of opacification with the SR between 0.93 and 0.97. The MTF performance was slightly decreased in IOLs #7 and #10, with the corresponding SR values of 0.79 and 0.75, respectively. IOL #6 demonstrated a lower optical quality (SR = 0.65), but the performance of IOL #5 was most affected, as the SR level was 0.57.

**Fig. 3:**
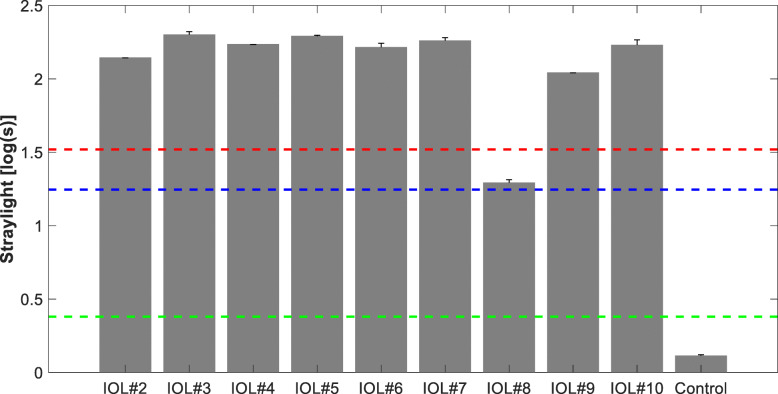
The straylight parameter of the explanted IOLs measured at a 3-mm aperture. The green and blue dashed lines are the theoretical levels of a normal 20-and 80-years-old crystalline lens. The red dashed line refers to the average straylight of an isolated cataractous lens. Error bars = standard deviation

Figure 3 presents the results of straylight measurements at 3 mm. We were unable to complete the assessment of IOL #1 as the straylight level was so extreme that the upper limit of the C-Quant was reached. Consequently, the reliability parameter esd was insufficient. The average straylight value of the remaining IOLs was 150.2 ± 56.3 deg^2^/sr. All the opacified IOLs but one showed the straylight level that was higher than that of a lens with cataract demonstrating a broad range of straylight elevation (from 3- to 6-fold increase compared to cataract). Although IOL #8’s straylight was lower than that of cataract, it was comparable to that of the 80-year-old crystalline lens. The control was almost free of scattering effects, which were negligible and below the level reported for a healthy twenty-year-old crystalline lens.

## Discussion

We demonstrated that primary IOL opacification has the potential to increase HOAs. Although the increase seems significant when compared with that of a clear lens, the impact on the MTF-derived optical quality was limited or not observed all. Straylight elevation, however, was severe and exceeded the level reported in patients with cataract.

HOAs have often been assessed clinically to compare the performances of different IOL models. Choi and Tchah compared individual Zernike modes in patients with three models of spherical IOLs and phakic controls [14]. They used a commercial Hartmann-Shack aberrometer with a wavelength of 780 nm and measured HOAs at a 4-mm non-dilated pupil and found the RMS of coma-like and spherical-like aberrations in pseudophakic eyes ranged, on average, between 0.123 μm to 0.146 μm, and 0.075 μm to 0.082 μm, respectively [14]. In our study, the RMS of coma aberrations in opacified IOLs was much lower, and that was 0.035 μm at 4 mm; which cannot cause a visual disturbance. The level of spherical aberration appears to be similar as in studied spherical IOLs, which ranged from 0.022 μm to 0.116 μm, and showed a clear dependence on the refractive power, as the highest spherical aberration value measurable at 4 mm was found in a 25D lens. Later, Kasper et al. applied Choi and Tchah’s aberrometry technique for a contralateral comparison of HOAs in pseudophakic patients with a spherical IOL in one eye and an IOL with spherical-aberration correction in the fellow eye [15]. Measurements were achieved following the instillation of a mydriatic agent, and Zernike coefficients were rescaled to various pupil sizes, including those used in our study. The median HOA RMS at 3 mm reported by Kasper et al. was approx. 0.07 μm [15], which was slightly higher than found in our explanted IOLs. At 4 mm, the median RMS was between 0.16 μm and 0.17 μm [15], which is about two-fold higher than that presented in Table 3. Although the difference in HOAs between the spherical and aspheric models was statistically significant at 6 mm, VA did not differ between the groups [15]. In another paper, Muñoz et al. also assessed patients implanted with spherical and aspheric IOLs and compared their HOAs [16]. Ocular HOAs were measured with a Hartmann-Shack device at 840 nm after pupil dilatation and recalculated for 4 mm [16]. They reported the higher-order RMS of approx. 0.22 μm and 0.26 μm for the aspheric technology and 0.33 μm and 0.49 μm, for two spherical IOLs used in that study. Despite that, visual function tests did not reveal significant differences between the IOL groups [16]. The SR derived from clinically measured HOAs ranged approximately from 0.04 to 0.16 at 4 mm [16]. By contrast, the lowest SR obtain in our study was 0.57. We also found that the total HOAs level in opacified IOLs is 3- to 5.6-fold lower than that found by Muñoz et al. Thus, we expect that the potential of HOAs measured in opacified lenses to affect VA is even more limited. Clinical data presented in Table 1 appear to confirm this assumption, as only in one known case, VA improved after IOL explantation. However, these VA results need to be interpreted with caution, as they had been collected at various centers in a non-standardized manner. More research is needed to assess IOL opacification’s impact on VA in a prospective clinical study.

Our results indicate that HOAs in opacified IOLs is an unlikely cause of dysphotopsia in patients with IOL calcification, as the measured values were below the level of a normal pseudophakic eye [17]. While the impact of increased turbidity on wave aberrations has been measured in cases with lenticular and posterior capsule opacification (PCO), there is as yet no study of HOAs in patients with calcified IOLs. Sachdev et al. recruited patients with early cataract to assess their aberrations [12]. Subjects’ pupils were dilated, and the wavefront was measured using a near-infrared Hartmann-Shack sensor [12]. Zernike modes were reported only for a 6-mm pupil [12]. They found that patients with cortical cataract tend to have increased coma and trefoil, and in the eyes with nuclear cataract trefoil predominated. In addition, their patients showed changes in spherical aberrations compared to healthy controls. Primary spherical aberration was also increased in cases with PCO, as reported by Rozema et al. [18] They measured HOAs in patients before and after neodymium: YAG laser capsulotomy using a ray-tracing aberrometer with 785-nm light. In that study, patients had either a hydrophilic or hydrophobic spherical IOL [18]. Tropicamide was used for pupil dilation prior to wave aberrations measurements; the analysis was performed at 5 mm [18]. In the pooled analysis for both groups, they found a significant decrease in the total HOAs after the YAG capsulotomy. However, the comparison within the hydrophilic and hydrophobic groups demonstrated that this reduction was significant only in the hydrophobic group [18]. Rozema et al. found a sizeable change in primary astigmatism following YAG capsulotomy, although the difference was not statistically significant [18]. In the current study, we also showed that ${\mathrm{Z}}_2^{-2}$ and ${\mathrm{Z}}_2^2$ increase in the presence of IOL opacification, which were the dominant aberrations found in our test lenses. It is not feasible, however, to precisely infer the impact of IOL turbidity on spherical aberration as measured here, as each test opacified IOL would need to be matched with a clear lens of the same power and optical design. Typically, a clear rotationally-symmetric and well-centered IOL is free from HOAs, except for spherical aberration. The measurements of the control lens confirm that the amplitude of astigmatism, coma, and trefoil aberrations are minimal, with the RMS range between 0.001 μm and 0.004 μm. Non-zero RMS values may stem from the alignment of the control lens, as tilt and decentration induce wave aberrations [30]. The levels found in the explanted lenses were much higher, by one order of magnitude, indicating that IOL opacification increases HOAs, particularly astigmatism and coma. The presence of these (micro) aberrations, as referred by van den Berg [21], may explain relatively uncompromised MTF performance in lenses with a homogenous and localized type of opacification reported in the literature [7, 31, 32]. However, sporadically, a dramatic reduction of the MTF has also been seen [4, 33], which may result from the level of wave aberrations not observed in our study.

In contrast to the small effect that IOL opacification had on HOAs and thus the MTF, straylight elevation was extreme in our test lenses. Of the nine IOLs that we were able to measure, eight showed the straylight value higher than that of the cataractous lens. Given extreme straylight values, one may expect that the MTF loss is sizable. In this study, such an effect was not observed, though. The reason for that might be that the measurement of aberrations derives from the centroids’ location of the Hartmann–Shack microlens system. The wavefront calculation assumes all those areas to contribute equally. However, considering high straylight values and inhomogeneity over the pupil plane, this might not be the case. Thus, for the MTF or the PSF derived from the wavefront (centroid location), scattered light may have little or no effect. For that reason, we assessed HOAs and straylight using different techniques. Although the straylight impact on the MTF might be underestimated, the Hartmann–Shack technique provides a reliable estimation of wavefront aberrations, indicating that homogeneous opacification has limited effect on HOAs.

Although we were not informed about patient-reported disphotopsia prior to explanation, one may expect that such high straylight must have caused glare symptoms [34]. Besides the characteristic complaint of being blinded while driving by oncoming car headlights, symptoms may also include hazy vision and hampered face- and color-recognition [26, 34, 35]. A large population study of 2422 European drivers assessed visual function in relation to lens opacity [36]. As a result, Michael et al. set a cut-off straylight value of 1.4 log(s) (s = 25.1 deg^2^/sr) to determine the level above which a person is deemed unfit to drive [36]. All studied IOLs, but one exceeded that level by a factor of 5 to 8; thus, IOL opacification not only affects the visual quality but may also impede driving ability and increase the risk of road accidents [36, 37]. Consequently, the 1.4 log(s) cut-off may be considered a safety reason for explantation of an opacified IOL among active drivers. In one case, light scattering intensity was slightly above the normative limits of a healthy 80-year-old crystalline lens. Although the straylight parameter was below the safety limit (s = 19.6 deg^2^/sr), it is still elevated compared to a normal pseudophakic eye of a similar age (s = 15.5 deg^2^/sr) [38]. Particularly in younger patients, this straylight level and the resultant dysphotopsia may require IOL exchange [31]. In a recent study, our group analyzed a series of opacified Mplus IOLs (Oculentis GmbH, Germany) of the same manufacturer as the test lenses we studied [7]. Those segmental, multifocal IOLs are made of identical hydrophilic material and had developed a similar pattern of homogenous opacification to the lenses in our study. Straylight values were determined using the C-Quant with the adaptation for in vitro IOL assessment, but the MTF performance was derived from the measurement of the line spread function [7]. We found an average straylight parameter of 170.1 ± 71.5 deg^2^/sr (higher than that found here), but MTF effects were also scarce [7]. However, homogenous calcification may, in rare cases, induce much higher straylight levels. Van der Meulen et al. assessed the straylight of explanted Aqua-Sense IOLs (Ophthalmic Innovation International, Inc., USA) using a goniometer light scattering setup [2]. They reported a straylight value of 794 deg^2^/sr in one opacified sample at 7.5°, which falls into the angular range of the C-Quant device [2]. One may wonder whether our IOL#1, which was so severely opacified that we could not measure it, would not reach a similar level.

## Conclusions

We demonstrated that IOL opacification gives rise to HOAs. However, the level of produced aberrations is low compared to that found in healthy pseudophakic eyes. In three IOLs, we could not measure the RMS level reliably at all apertures due to severe opacification. Thus, we cannot rule out a significant increase in HOAs in such cases in vivo. Although increased HOAs resulted in a decrease in the MTF performance in half of the studied IOLs, the resulting SR was still higher than that reported in healthy eyes. Given that, we expect that micro-aberrations found in our study have a low potential to contribute to dysphotopsia. On the other hand, light scattering effects were evident, as we saw on average, a nearly five-fold increase from the level for a crystalline lens with cataract. Increased straylight may result in disability glare that affects patients’ daily activities despite preserving good high-contrast acuity. Besides straylight measurements, the addition of low-contrast VA tests or determining a CS function under glare conditions may provide a further understanding of IOL opacification’s impact on visual performance.

## Data Availability

The datasets used and analyzed for the present study are available from the corresponding author upon reasonable request.

## Abbreviations

CS: Contrast sensitivity
D: Diopter
HOAs: Higher-order aberrations
IOL: Intraocular lens
MTF: Modulation transfer function
PSF: Point spread function
RMS: Root mean square
SR: Strehl ratio
VA: Visual acuity

## References

[1] Izak AM, Werner L, Pandey SK, Apple DJ. Calcification of modern foldable hydrogel intraocular lens designs. Eye. 2003;17(3):393–406. 10.1038/sj.eye.6700341 12724703

[2] van der Meulen IJ, Porooshani H, Van den Berg TJ. Light-scattering characteristics of explanted opacified Aquasense intraocular lenses. Br J Ophthalmol. 2009;93(6):830–2. 10.1136/bjo.2008.151324 19060012

[3] Khoramnia R, Salgado JP, Auffarth GU, Schmidt S, Wegner A, Kobuch KA, et al. Opacification of a hydrophilic intraocular lens 4 years after cataract surgery. A biomaterial analysis. Ophthalmologe. 2012;109(5):483–6. 10.1007/s00347-011-2487-6 22415452

[4] Tandogan T, Khoramnia R, Choi CY, Scheuerle A, Wenzel M, Hugger P, et al. Optical and material analysis of opacified hydrophilic intraocular lenses after explantation: a laboratory study. BMC Ophthalmol. 2015;15:170. 10.1186/s12886-015-0149-1 PMC4659174 26606985

[5] Gurabardhi M, Häberle H, Aurich H, Werner L, Pham DT. Serial intraocular lens opacifications of different designs from the same manufacturer: clinical and light microscopic results of 71 explant cases. J Cataract Refract Surg. 2018;44(11):1326–32. 10.1016/j.jcrs.2018.07.026 30279087

[6] Neuhann T, Yildirim TM, Son HS, Merz PR, Khoramnia R, Auffarth GU. Reasons for explantation, demographics, and material analysis of 200 intraocular lens explants. J Cataract Refract Surg. 2020;46(1):20–6. 10.1016/j.jcrs.2019.08.045 32050228

[7] Yildirim TM, Labuz G, Khoramnia R, Son HS, Schickhardt SK, Lieberwirth I, et al. Impact of primary calcification in segmented refractive bifocal intraocular lenses on optical performance including straylight. J Refract Surg. 2020;36(1):20–7. 10.3928/1081597X-20191119-01 31917847

[8] Neuhann IM, Kleinmann G, Apple DJ. A new classification of calcification of intraocular lenses. Ophthalmology. 2008;115(1):73–9. 10.1016/j.ophtha.2007.02.016 17498804

[9] Fernández-Buenaga R, Alió JL. Intraocular lens explantation after cataract surgery: indications, results, and explantation techniques. Asia Pac J Ophthalmol (Phila). 2017;6(4):372–80. 10.22608/APO.2017181 28780780

[10] Werner L, Wilbanks G, Nieuwendaal CP, Dhital A, Waite A, Schmidinger G, et al. Localized opacification of hydrophilic acrylic intraocular lenses after procedures using intracameral injection of air or gas. J Cataract Refract Surg. 2015;41(1):199–207. 10.1016/j.jcrs.2014.10.025 25465216

[11] Marsack JD, Thibos LN, Applegate RA. Metrics of optical quality derived from wave aberrations predict visual performance. J Vis. 2004;4(4):322–8. 10.1167/4.4.8 15134479

[12] Sachdev N, Ormonde SE, Sherwin T, McGhee CN. Higher-order aberrations of lenticular opacities. J Cataract Refract Surg. 2004;30(8):1642–8. 10.1016/j.jcrs.2004.02.048 15313285

[13] Thibos LN, Hong X, Bradley A, Applegate RA. Accuracy and precision of objective refraction from wavefront aberrations. J Vis. 2004;4(4):329–51. 10.1167/4.4.9 15134480

[14] Choi J, Kim TI, Tchah H. Comparison of wavefront aberration after cataract surgery with acrylic intraocular lens implantation. J Cataract Refract Surg. 2005;31(2):324–9. 10.1016/j.jcrs.2004.06.021 15767153

[15] Kasper T, Bühren J, Kohnen T. Intraindividual comparison of higher-order aberrations after implantation of aspherical and spherical intraocular lenses as a function of pupil diameter. J Cataract Refract Surg. 2006;32(1):78–84. 10.1016/j.jcrs.2005.11.018 16516783

[16] Muñoz G, Albarrán-Diego C, Montés-Micó R, Rodríguez-Galietero A, Alió JL. Spherical aberration and contrast sensitivity after cataract surgery with the Tecnis Z9000 intraocular lens. J Cataract Refract Surg. 2006;32(8):1320–7. 10.1016/j.jcrs.2006.02.055 16863968

[17] Sharma M, Wachler BS, Chan CC. Higher order aberrations and relative risk of symptoms after LASIK. J Refract Surg. 2007;23(3):252–6. 10.3928/1081-597X-20070301-07 17385290

[18] Rozema JJ, Koppen C, de Groot V, Tassignon MJ. Influence of neodymium: YAG laser capsulotomy on ocular wavefront aberrations in pseudophakic eyes with hydrophilic and hydrophobic intraocular lenses. J Cataract Refract Surg. 2009;35(11):1906–10. 10.1016/j.jcrs.2009.06.033 19878822

[19] Chalita MR, Xu M, Krueger RR (2003). Correlation of aberrations with visual symptoms using wavefront analysis in eyes after laser in situ keratomileusis. J Refract Surg.

[20] Koh S. Mechanisms of visual disturbance in dry eye. Cornea. 2016;35(Suppl 1):S83–S88. 10.1097/ICO.0000000000000998 27583799

[21] van den Berg TJ. The (lack of) relation between straylight and visual acuity. Two domains of the point-spread-function. Ophthalmic Physiol Opt. 2017;37(3):333–41. 10.1111/opo.12368 28271538

[22] Noll RJ. Zernike polynomials and atmospheric turbulence. J Opt Soc Am A. 1976;66(3):207–11.

[23] Łabuz G, Vargas-Martín F, van den Berg TJ, López-Gil N. Method for in vitro assessment of straylight from intraocular lenses. Biomed Opt Express. 2015;6(11):4457–64. 10.1364/BOE.6.004457 PMC4646552 26601008

[24] Łabuz G, Papadatou E, Vargas-Martín F, López-Gil N, Reus NJ, van den Berg TJTP. Validation of a spectral light scattering method to differentiate large from small particles in intraocular lenses. Biomedical Optics Express. 2017;8(3):1889–94. 10.1364/BOE.8.001889 PMC5480586 28663871

[25] Franssen L, Coppens JE, van den Berg TJ. Compensation comparison method for assessment of retinal straylight. Invest Ophthalmol Vis Sci. 2006;47(2):768–76. 10.1167/iovs.05-0690 16431978

[26] van den Berg TJ, Franssen L, Kruijt B, Coppens JE. History of ocular straylight measurement: a review. Z Med Phys. 2013;23(1):6–20. 10.1016/j.zemedi.2012.10.009 23182462

[27] Vos J, Van den Berg T (1999). Report on disability glare. CIE collection.

[28] van den Berg TJ. Analysis of intraocular straylight, especially in relation to age. Optom Vis Sci. 1995;72(2):52–9. 10.1097/00006324-199502000-00003 7753528

[29] de Wit GC, Franssen L, Coppens JE, van den Berg TJ. Simulating the straylight effects of cataracts. J Cataract Refract Surg. 2006;32(2):294–300. 10.1016/j.jcrs.2006.01.048 16565008

[30] Lawu T, Mukai K, Matsushima H, Senoo T. Effects of decentration and tilt on the optical performance of 6 aspheric intraocular lens designs in a model eye. J Cataract Refract Surg. 2019;45(5):662–8. 10.1016/j.jcrs.2018.10.049 30876781

[31] Łabuz G, Yildirim TM, van den Berg TJ, Khoramnia R, Auffarth GU. Assessment of straylight and the modulation transfer function of intraocular lenses with centrally localized opacification associated with the intraocular injection of gas. J Cataract Refract Surg. 2018;44(5):615–22. 10.1016/j.jcrs.2018.01.033 29891155

[32] Yildirim TM, Auffarth GU, Łabuz G, Bopp S, Son HS, Khoramnia R. Material analysis and optical quality assessment of opacified hydrophilic acrylic intraocular lenses after pars plana vitrectomy. Am J Ophthalmol. 2018;193:10–9. 10.1016/j.ajo.2018.06.002 29890164

[33] Giers BC, Tandogan T, Auffarth GU, Choi CY, Auerbach FN, Sel S, et al. Hydrophilic intraocular lens opacification after posterior lamellar keratoplasty-a material analysis with special reference to optical quality assessment. BMC Ophthalmol. 2017;17(1):150. 10.1186/s12886-017-0546-8 PMC5568293 28830376

[34] van den Berg TJ. On the relation between glare and straylight. Doc Ophthalmol. 1991;78(3–4):177–81. 10.1007/BF00165678 1790738

[35] Elliott DB, Hurst MA, Weatherill J. Comparing clinical tests of visual function in cataract with the patient's perceived visual disability. Eye (Lond). 1990;4(Pt 5):712–7. 10.1038/eye.1990.100 2282946

[36] Michael R, van Rijn LJ, van den Berg TJ, Barraquer RI, Grabner G, Wilhelm H, et al. Association of lens opacities, intraocular straylight, contrast sensitivity and visual acuity in European drivers. Acta Ophthalmol. 2009;87(6):666–71. 10.1111/j.1755-3768.2008.01326.x 18786129

[37] Bal T, Coeckelbergh T, Van Looveren J, Rozema JJ, Tassignon MJ. Influence of cataract morphology on straylight and contrast sensitivity and its relevance to fitness to drive. Ophthalmologica. 2011;225(2):105–11. 10.1159/000317076 20881445

[38] Łabuz G, Reus NJ, van den Berg TJ. Ocular straylight in the normal pseudophakic eye. J Cataract Refract Surg. 2015;41(7):1406–15. 10.1016/j.jcrs.2014.10.035 26287879

